# Pattern of antibiotic use among children caregivers: a cross-sectional study

**DOI:** 10.1186/s42506-024-00176-6

**Published:** 2024-12-05

**Authors:** Doaa S. Ahmed, Asmaa M. AboElela, Samar S. Ismail, Zeinab E. Hammour, Rasha A. Fawaz, Marwa E. Abdelmoniem

**Affiliations:** 1 Public Health, Community Medicine and Occupational Medicine Department, Faculty of Medicine for Girls, Al-Azhar University, Cairo, Egypt; 2 Pediatric Medicine Department, Faculty of Medicine for Girls, Al-Azhar University, Cairo, Egypt

**Keywords:** Antibiotics, Misuse, Parents of children

## Abstract

**Background:**

Antibiotic resistance, a major global health concern, is primarily caused by the irrational use of antibiotics. Parents play a crucial role in antibiotic use by children, directly impacting their clinical outcomes. This study aimed to assess the pattern of antibiotic use among parents and its related factors.

**Methods:**

A cross-sectional study was conducted among 400 parents randomly selected from the pediatric outpatient clinics of Al-Zahraa University Hospital, Cairo. Data were collected using a structured interview questionnaire about sociodemographic data, antibiotic use patterns, parental knowledge and attitudes, common sources of information, and reasons for self-prescribing antibiotics.

**Results:**

Out of the 400 parents surveyed, 87.7% were mothers with a mean age of 31.9 ± 7 years, 76% were highly educated, almost half were not working, 63% came from urban areas, and 95.2% had sufficient income. As for their children, 57.5% had male children; their average age was 5.5 ± 3.7 years. The misuse of antibiotics was prevalent among 37.2% (95% *CI* = 32.5–42.2%) of parents. When assessing parents’ knowledge and attitude towards antibiotic use, 30.2% (95% *CI* = 25.8–35.0%) had good knowledge which was significantly more common among highly educated, working, and high-income parents, while the majority (72%, 95% *CI* = 67.3–76.3%) had a positive attitude. Having more and older children significantly increased the probability of antibiotic misuse, while a higher level of parental education and a positive attitude decreased the likelihood of such misuse (odds ratio (OR) was 1.31, 1.09, 0.52, and 0.11, respectively). Most parents obtained their information about antibiotics from physicians (92%) followed by experience from previous prescription (58.5%). Moreover, among inappropriate users, previous prescriptions and the parent’s perception of the child’s illness as a nonurgent condition were the most frequently cited reasons for the parents’ self-prescription of antibiotics (76.5% and 69.1%, respectively).

**Conclusion:**

Approximately, one-third of surveyed parents demonstrated an inappropriate use of antibiotics. Despite possessing a highly positive attitude and a reasonable level of knowledge about antibiotics, parents often underestimate the potential adverse effects. Tailored measures to promote appropriate antibiotic use are therefore needed to combat the problem of antibiotic resistance.

## Introduction

Although antibiotics are one of the greatest inventions and among the most affordable drugs that save millions of lives from various infections [1], antimicrobial resistance (AMR) is fast becoming one of the biggest threats to public health. The World Health Organization (WHO) defines AMR as the bacterial ability to no longer respond to the initially powerful antibiotics used to treat such infections [2]. In addition to its natural occurrence, the misuse of antibiotics in humans and animals is accelerating the process and spreading AMR around the world [3].

Irrational use of antibiotics includes excessive or inadequate prescribing, inappropriate self-medication, skipping doses, reuse of leftover antibiotics, inappropriate selection of antibiotics, harmful combination or inappropriate dosing regimen, excessive duration of medication, incomplete treatment regimens, frequent non-indicated use of antibiotics, and noncompliance with clinical guidelines [4]. Irrational use of antibiotics has been observed to be ubiquitous in low- and middle-income countries, where prescribing without laboratory evidence and nonprescription sales are commonly practiced [5].

During the COVID pandemic, governments around the world were forced to implement restrictive measures such as lockdowns and social distancing. Many people turned to self-medication due to the extreme difficulties in accessing healthcare services [6]. Vaccine shortages, inadequate treatment, widespread fear of viruses, and infodemics in digital and social media have increased self-medication practices [7]. A multinational cross-sectional study conducted in 10 Arab countries during this period found that Egypt had the highest prevalence of self-medication practices (72.1%), with antibiotics accounting for 43.3% of the drugs used for self-medication [8].

The harmful effects of antibiotic misuse range from increased morbidity and mortality rates, higher healthcare costs, and patients’ mistrust of physicians and health authorities. Most critically however is its contribution to the emergence of antibiotic resistance, which is a significant public health problem worldwide that is not limited to developing countries [9, 10]. Without effective action, the lethal impact of AMR is estimated to increase further, reaching 10 million deaths per year worldwide by 2050 [11]. Additionally, the economic impact may reach US $1 trillion annually by 2050 [12, 13].

The inappropriate use of antibiotics in the general population has been linked to inadequate knowledge and attitude [14–16]. Considering that children are more vulnerable to respiratory tract infections and other infections, their parents or guardians play a crucial role in the treatment of their illnesses and have the choice of when and how to administer antibiotics. It has been reported that many parents give antibiotics to their children to treat the symptoms of cold, flu, fever, and respiratory illnesses [17].

Numerous factors such as overprescription by physicians, easy availability of antibiotics for self-medication, and inadequate parental awareness, perception, and behavior regarding antibiotic use all contribute to the inappropriate use of antibiotics among children [18, 19]. Also, pressure on physicians to prescribe more antibiotics to satisfy parents and meet their high expectations for symptom relief and quick recovery of their children has increased [20].

Understanding the knowledge, attitude, and behaviors of parents and identifying the key factors that influence their decision-making related to antibiotic use by their children are crucial for planning effective interventions for improving parents’ practices. The current study aims to identify the patterns of antibiotic use among caregivers of children and quantify the prevalence of misuse. It also aims to identify the potential factors contributing to inappropriate antibiotic use among caregivers.

## Methods

### Study design and setting

A cross-sectional study was conducted between September 2022 and July 2023 among parents or caregivers who sought medical advice for their children at the pediatric outpatient clinics of Al-Zahraa University Hospital, a unique hospital affiliated with the Faculty of Medicine for Girls (FMGs) at Al-Azhar University in Cairo.

### Sample size and sampling technique

The sample size was calculated using the following formula: (*n* = [(Zα/2)^2^*P(1-P)]/d^2^), where Zα/2 = 1.96 considering a confidence level of 95%, a marginal error (d) of 5%, and the estimated prevalence of antibiotic misuse among Egyptian children (50%) based on previous studies [21, 22]. The minimum calculated sample size was 385, but it was rounded up to 400 participants who were recruited through systematic random sampling from parents or caregivers of children attending the pediatric clinic on randomly selected days.

### Data collection tools

An interview questionnaire was used, which took an estimated 20 min to complete. It was structured to collect the following data:

1. The sociodemographic profile of the parents or responsible caregivers which included the following: age, place of residence, education, occupation, income, family type, and number of children, in addition to the age and gender of the child who came to the outpatient clinic.
2. The pattern of antibiotic use by parents was determined, with responses ranging from always to never. During analysis, we grouped always and often together and scored them as (1), while sometimes, rarely, and never were grouped and scored as (0). Based on the total score, practices were categorized into misuse (scores 0–79%) and appropriate use (scores 80–100%).
3. The potential factors contributing to parental antibiotic misuse were explored by assessing parental antibiotic knowledge and attitude.
  Knowledge of the trade names of antibiotics, their possible side effects, their role, and their resistance was assessed with the answers “yes,” “no,” or “I don’t know.” Each correct answer was scored as 1, while incorrect or uncertain answers were scored as 0. Participants’ overall knowledge was then rated as either poor (0–49%), moderate (50–79%), or good (80–100%).
  Parents’ attitudes towards antibiotic use, safety, and medical prescriptions were rated using a 5-point Likert scale from “strongly disagree” = 1 to “strongly agree” = 5. During the analysis, we summarized the responses by combining “strongly agree” with “agree” and “strongly disagree” with “disagree.” Attitudes were categorized as negative (0–49%), neutral (50–79%), and positive (80–100%).

This scale was developed on the basis of a literature research [17, 23] in English. It was translated and adapted cross-culturally, with the forward translation into Arabic done by a bilingual professional translator in English, followed by a backward translation to check that the meaning of the items was retained. An expert committee composed of two public health professionals and a research methodologist reviewed the clarity of the format and appropriateness of the content, and the necessary adjustments were made based on their recommendations. In addition, the preliminary Arabic version of the questionnaire was pilot-tested with 5% of the sample (which was excluded from the final analysis) to check the clarity of the questions and to estimate the time needed to complete the questionnaire, with no changes to the instruments. In addition, Cronbach’s alpha was used to quantify internal consistency. The results showed that knowledge, attitude, and practice had values of 0.83, 0.78, and 0.77, respectively.

The scores for knowledge, attitude, and practice were calculated as a proportion of responses to the total questions asked in each category and then classified according to Mutagonda et al. [17].

4. The most common reasons for parental self-medication of antibiotics and sources of information about antibiotics.

### Statistical analysis

Data were analyzed using the IBM Statistical Package for Social Science (SPSS) version 16 [24]. Quantitative data were expressed as mean ± SD and range, while qualitative data were expressed as frequencies and percentages. The chi-square test was used to compare qualitative variables. A binary logistic regression analysis was used to identify factors associated with parents’ misuse of antibiotics in children. Odds ratio and 95% confidence intervals were reported, and a *p*-value < 0.05 was considered statistically significant.

## Results

The current study included 400 parents; 87.7% were mothers, and 57.5% of their children were male; the age of the children ranged from 5 days to 17 years, with a mean age of 5.5 ± 3.7 years. Table 1 shows that the mean age of the parents was 31.9 ± 7 years. Although 76% had a higher level of education, almost half (47%) were not employed. About two-thirds (63%) were from urban areas. Most of parents (95.2%) reported having sufficient income for at least necessary needs, 73.5% had two or more children, and more than half (54.5%) lived in a nuclear family.

**Table 1:** Sociodemographic characteristics of the studied parents at Al-Zahraa University Hospital, Cairo, during the year 2023

Variables	Categories	*N* = 400	**%**
**Age of parents (years): mean ± SD**	31.9 ± 7		
**Parents’ education**			
Secondary and below	96		24.0
Above secondary	304		76.0
**Parents’ working status**			
Employed	172		43.0
Self employed	40		10.0
Not working	188		47.0
**Place of residence**			
Rural	148		37.0
Urban	252		63.0
**Family monthly income**			
Not enough	19		4.8
Enough for necessary needs only	122		30.5
Enough for necessary needs and emergencies	213		53.2
Enough and extra	46		11.5
**Number of children**			
One child	106		26.5
Two children	167		41.8
Three or more children	127		31.7
**Family type**			
Nuclear	218		54.5
Extended	148		37.0
Single parent	34		8.5

**Fig. 1:**
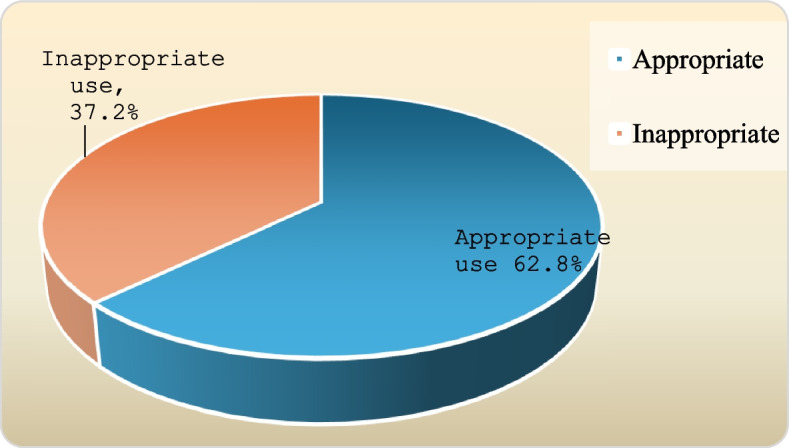
Prevalence of antibiotic use among the studied group

Figure 1 illustrates that 37.2% of the parents were abusing antibiotics

Of all parents surveyed, 30.2% had good overall knowledge. In terms of sub-domains of knowledge, 53.5%, 42.8%, 43.4%, and 41.5% had good knowledge of the trade name of the antibiotic, the role of the antibiotic, antibiotic resistance, and the side effects of the antibiotic respectively (Table 2).

**Table 2:** Level of parents’ knowledge about antibiotic use

Knowledge level	Poor	Moderate	Good
Domains	*N* (%)	*N* (%)	*N* (%)
**Total knowledge score**	42 (10.6)	237 (59.2)	121 (30.2)
**Antibiotics trade names**	76 (19.0)	110 (27.5)	214 (53.5)
**Role of antibiotics**	51 (12.8)	178 (44.4)	171 (42.8)
**Antibiotics resistance**	39 (9.8)	187 (46.8)	174 (43.4)
**Side effects of antibiotics**	124 (31.0)	110 (27.5)	166 (41.5)

**Fig. 2:**
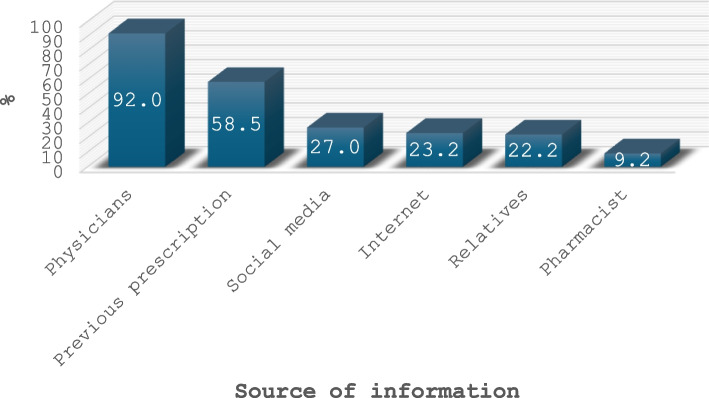
Source of information about antibiotic among the studied parents

The vast majority (92%) of parents obtained their information from physicians, followed by experience from previous prescription (58.5%) (Fig. 2).

Relating the parents’ knowledge levels to their sociodemographic data revealed that the majority of those with good knowledge had a high educational level and enough/extra monthly income (86.8 and 81.0%, respectively). In addition, almost two-thirds of parents with good knowledge lived in urban areas and were employed (62.0 and 60.38%, respectively), while 54.5% lived in nuclear families. The differences were statistically significant (*p* < 0.05) only in terms of parents’ education, working status, and income (Table 3).

**Table 3:** Association of demographic factors with parents’ knowledge levels of antibiotic use

Knowledge level	Poor	Moderate	Good	Sig. test *p*-value
**General characteristics**	*N* = 42 (%)	*N* = 237 (%)	*N* = 121 (%)	
**Parent education**				
Secondary and below	28 (66.7)	52 (21.9)	16 (13.2)	*p* < 0.05*
Above secondary	14 (33.3)	185 (78.1)	105 (86.8)	
**Family monthly income**				
Enough necessary needs only	28 (66.7)	90(37.9)	23(19.0)	*p* < 0.05*
Enough and extra	14 (33.3)	147(62.1)	98 (81.0)	
**Parents residence**				*p* = 0.9
Rural	16 (38.1)	86 (36.3)	46 (38.0)	
Urban	26 (61.9)	151 (63.7)	75 (62.0)	
**Family type**				*p* = 0.1
Nuclear	17 (40.5)	135 (57.0)	66 (54.5)	
Extended	17 (40.5)	84 (35.4)	47 (38.8)	
Single parent	8 (19)	18 (7.6)	8 (6.7)	

^*^Statistically significant difference

Table 4 shows parents’ attitudes towards antibiotic use: 72% had a positive overall attitude, with 79.6%, 55.6%, and 42.8% having a positive attitude towards antibiotic prescribing, antibiotic use, and antibiotic safety and resistance, respectively.

**Table 4:** Parents’ attitudes towards antibiotics use

Attitude grading	Negative No. (%)	Neutral No. (%)	Positive No. (%)
Domains			
**Total attitude score**	9(2.2)	103 (25.8)	288 (72.0)
**Attitude towards doctor’s prescription of antibiotics**	37 (9.2)	45 (11.2)	318 (79.6)
**Attitude towards antibiotic use**	17 (4.2)	161 (40.2)	222 (55.6)
**Attitude towards safety and resistance**	13 (3.2)	216 (54.0)	171 (42.8)

Among those with antibiotic misuse, only 19.5% had good knowledge, and less than half (45%) had a positive attitude, compared to 36.7% and 88% respectively among appropriate antibiotic users with statistically significant differences (*p*-value < 0.05) (Table 5).

**Table 5:** Level of knowledge and attitude in relation to antibiotic use among the studied parents

**Antibiotic use** **Factors**	**Inappropriate users *N* = 149**		**Appropriate users *N* = 251**		**Significant test** ***p*-value**
	**No.**	**%**	**No.**	**%**	
**Knowledge level**					*p* < 0.05*
Poor	23	15.4	19	7.5	
Moderate	97	65.1	140	55.8	
Good	29	19.5	92	36.7	
**Attitude categories**					*p* < 0.05*
Negative	9	6.0	0	0.0	
Neutral	73	49.0	30	12.0	
Positive	67	45.0	221	88.0	

^*^Statistically significant difference

The logistic regression analysis in Table 6 detected that the likelihood of parents’ misuse significantly increases with having more children, and with an increase in the child’s age, while it decreases with higher parents’ education and positive attitude (*OR* = 1.309, 1.092, 0.522, and 0.111 were recorded respectively).

**Table 6:** Logistic regression of factors associated with antibiotic misuse among the studied parents

Factors	*B*	Wald	Sig.	OR	95.0% CI for OR	
					**Lower**	**Upper**
**Number of children**	0.269	4.605	0.032*	1.309	1.024	1.673
**Child age**	0.088	6.162	0.013*	1.092	1.019	1.171
**Parents education level**	− 0.650	5.050	0.025*	0.522	0.296	0.920
**Attitude towards antibiotic use**	− 2.200	64.415	0.000*	0.111	0.065	0.190
**Knowledge about antibiotic use**	− 0.123	0.311	0.577	0.884	0.574	1.363
**Constant**	5.676	39.553	0.000	291.711		

^*^Statistically significant

**Fig. 3:**
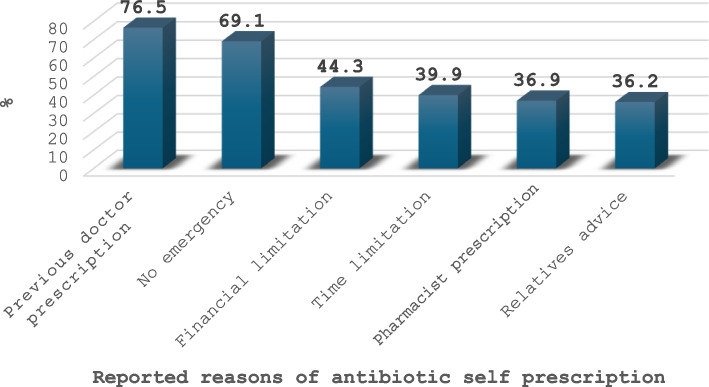
Reported reasons for antibiotic’ self-prescription among the studied misusers

Figure 3 illustrates that previous antibiotic prescriptions (76.5%) and parents’ belief that their child’s illness is not an emergency (69.1%) were the main reasons for the self-prescribing behavior. Other reasons were financial constraints (44.3%), lack of time (39.9%), prescription by a pharmacist (36.9%), and advice from a relative (36.2%).

## Discussion

Infection is a major health concern in developing low-resource countries that represents a serious leading cause of morbidity and mortality, especially in children. To cope with financial life challenges, parents try to save both money and time by using their knowledge and experience to treat their children. Over-the-counter unrestricted access to antibiotics and the disseminated information about diseases and therapeutic modalities through the Internet encourage self-medication prescription by parents. As a consequence, inappropriate antibiotic use has emerged, contributing to the development of drug resistance. Misusing antibiotics is a multifactorial problem that has a negative impact on medical services and adversely affects clinical outcomes, especially in vulnerable populations such as children. The current study explored the pattern of antibiotic use by parents and the factors affecting this pattern in a sample of the Egyptian population.

The inappropriate use of antibiotics is not a hidden problem. Several studies have analyzed this problem with different results even within the same country. In the Egyptian population, the rate of antibiotic misuse ranged from 13 to 80% [25, 26]. This wide variation reflects the influence of sociodemographic and economic factors on antibiotic use patterns in the population. Previous studies have shown that lack of knowledge and low income were the two main factors contributing to inappropriate antibiotic use in developing countries. The highest rate of antibiotic misuse in the pediatric population was reported in the Middle East (34%), followed by Africa (22%) [27].

Among the studied children’s caregivers, the current study found 37.2% to be antibiotic misusers. Several studies evaluated parents´ antibiotic use in different areas of Egypt. Among Egyptian children, Osman et al. found that about 50% of mothers in Qena Governorate misused antibiotics [21], while Mohammed et al. found that more than two-fifths of parents in Alexandria used antibiotics without an acceptable prescription [22]. Other studies evaluated parents’ antibiotic use in different countries. Mallah et al. reported that 41% of Lebanese participants had at least one misuse behavior [28], and Alanazi et al. revealed that the prevalence of antibiotic misuse was 57.8% among children in Saudi Arabia [29]. Additionally, Mutagonda et al. found a much higher prevalence of misuse in Tanzania, where 82% of participants were inappropriate antibiotic users [17].

This wide range of values for reported misuse between studies could be attributed to differences in parents’ knowledge and attitude towards antibiotics. In our current study, 30.2% of the participants had good knowledge, and 72% had a positive attitude, which was greater than previous studies, where for instance only 10.9% had good knowledge and 16.4% had positive attitudes in the study in Tanzania [17].

The present study results revealed that a good knowledge level was significantly associated with higher education, having work, and having sufficient income. These findings are in accordance with studies in Tanzania and Bosnia [17, 30] but stand in contrast to the findings from Islam et al. who found that secondary education, not being employed, and having a low household income were associated with good knowledge in his study in Bangladesh [31].

Although the study participants had a good level of knowledge about the use and resistance of antibiotics, our research showed that 31% of them had poor knowledge about the side effects of antibiotics, and 54% had neutral attitudes towards the safety and resistance of antibiotics. Underestimation of the hazardous effects of antibiotics may contribute to their misuse by parents considering them as safe medications.

Furthermore, our study showed that 79.6% had positive attitudes towards doctor prescriptions of antibiotics. This positive attitude by the parents can potentially adversely affect physicians’ behaviors. Awad et al. reported that some physicians prescribe antibiotics to satisfy their patients and guarantee their visits in the future despite unnecessary indications for antibiotic use [32].

The current study demonstrated that a higher level of parents’ education and their positive attitude towards antibiotics were protective factors that decreased the proportion of inappropriate antibiotic use. These results corroborated those of earlier studies [22, 30, 33, 34].

In addition, having a higher number of children and an older age of children were associated with greater misuse of antibiotics among our participants. These strong associations reflect the financial burden, as more than half of our included parents relied on their previous experiences (58.5%) or previous prescriptions (76.5%) instead of visiting physicians.

The source of getting correct information about antibiotics is crucial for their appropriate use. In our study, most parents (92%) got their information from physicians, and more than half (58.5%) got their information based on their previous experience. Our findings were consistent with those of Awadh et al. [35] and Siddiqui et al. [36] in other investigations. However, these findings raise concerns that widespread prescriptions of antibiotics by medical staff may encourage parents to do the same the next time their children get ill. Evidence revealed that even among physicians and in hospital settings, there is a high rate of inappropriate use of antimicrobials [37]. Thus, we may be entering into a vicious cycle where inappropriate use of antibiotics leads to the emergence of drug resistance, which in turn leads to further prescription of more antibiotics and vice versa.

### Limitations of the study

This study has some limitations due to the relatively small sample of the population included and the cross-sectional design, which limits our ability to analyze the cause-and-effect relationship. We did not examine the consequences of inappropriate antibiotic use by parents, including overall financial costs, hazardous effects on children, and the emergence of antibiotic-resistant strains.

## Conclusion

The current study clarified that antibiotic misuse was prevalent among about one-third of the studied parents. A large proportion of parents had moderate to good knowledge level of antibiotic use, which is positively influenced by high education, income, and working status. The majority had a positive attitude. Inappropriate antibiotic use was significantly associated with lower education, negative attitudes, and having more and older children. Most parents received information about antibiotic use from physicians, followed by experience from previous prescription. Moreover, dependence on the previous prescription and considering the child’s illness as a non-emergent situation were the main reported reasons of antibiotic use without a prescription.

According to the study’s findings, it is crucial to improve parents’ understanding and encourage a positive attitude towards the correct use of antibiotics. This can be achieved by implementing community mobilization strategies, starting with strict government regulations that prevent the purchase of antibiotics without a physician's prescription and disseminating accurate information through social media platforms by emphasizing the continuous evolution of microbes, and the financial and health risks associated with improper medication use are vital. Leveraging new AI technologies to create engaging infographics and educational materials is an effective way to raise awareness among parents. Additionally, it is essential to encourage medical students and healthcare professionals to discuss the risks and benefits of antibiotics with parents and prescribe them only when necessary. Further research to evaluate these measures is recommended to achieve continuous improvement.

## Data Availability

Data are available from the corresponding author upon reasonable request.
